# Percutaneous trigeminal ganglion radiofrequency thermocoagulation alleviates anxiety and depression disorders in patients with classic trigeminal neuralgia: A cohort study

**DOI:** 10.1097/MD.0000000000005379

**Published:** 2016-12-09

**Authors:** YuanZhang Tang, Ling Ma, Na Li, Yuna Guo, Liqiang Yang, Baishan Wu, Jianning Yue, Qi Wang, Jingjie Liu, Jia-xiang Ni

**Affiliations:** a Department of Pain Management, Xuanwu Hospital, Capital Medical University, Beijing; b Department of Anesthesiology and Pain Management, Daqing Group Oilfield General Hospital, Heilongjiang, China.

**Keywords:** anxiety, depression, predictive factors, prevalence, radiofrequency thermocoagulation, trigeminal neuralgia

## Abstract

Trigeminal neuralgia (TN) is a neurological condition that presents as excruciating facial pain. Depression and anxiety are commonly associated with TN; however, anxiety and depression disorders in patients with TN and the effects of the various therapeutic strategies for TN on these disorders are not well studied.

To evaluate depression and anxiety in patients with trigeminal neuralgia (TN), identify factors that predict their occurrence and study the effect of the percutaneous trigeminal ganglion radiofrequency thermocoagulation (PRT) procedure for alleviating pain on depression and anxiety.

Patients with classic TN, who received PRT treatment, were consecutively recruited between October 2014 and October 2015. Severity of pain was determined using the visual analogue scale (VAS) score. Beck Depression Inventory-II (BDI) and Beck anxiety Inventory (BAI) were used to evaluate depression and anxiety disorders pre- and post-PRT. Medical, demographic, and psychosocial backgrounds were also assessed as predictive factors. A BDI score of ≥14 represented depression and BAI score of ≥45 represented anxiety. VAS, BDI, and BAI scores were collected at the time of admission and on the day of discharge.

Of the 167 patients who participated in the study, 121 (72.5%) had depression and 34 (20.4%) suffered anxiety. Pre-PRT procedure, female sex, age >50 years, ineffective treatment, and high pain intensity (VAS ≥7) predicted the development of depression and anxiety. Post-PRT procedure, all patients who experienced pain relief also reported amelioration of depression and anxiety.

A considerable percentage of patients with TN developed depression and anxiety. Patients who were female, older than 50 years, or suffered from failure treatment and severe pain (VAS>7), were at higher risk of depression and anxiety development. Complete alleviation of pain by using surgical PRT could immediately attenuate depressive and anxiety disorders associated with TN.

## 1 Introduction

Psychiatric comorbidities such as anxiety and depression disorders are highly prevalent in patients suffering from chronic pain. Depression is closely related to pain intensity and is 3 times more likely to occur in patients with chronic pain.^[1]^ Bair et al^[2]^ reported that 65% of patients with chronic pain have serious depression, and that treatment of depression and pain is necessary for improved outcomes. Castro et al^[3]^ reported that depression showed highest comorbidity with chronic pain, and 42% of patients with chronic pain suffered from depression. Kayhan et al^[4]^ found that 46.6% of patients with disc herniation had anxiety disorder.

Trigeminal neuralgia (TN) is a painful condition characterized by excruciating facial pain, and has a serious impact on quality of life. The pain can be triggered by normal daily activities such as touching the face, brushing teeth, chewing, or even talking. Patients with TN often develop a fear of triggering pain, which could lead to anxiety and depression, and considerably reduce their quality of life. Pharmacological research has led to considerable advancement in the treatment of TN. Anticonvulsive drugs such as carbamazepine often serve as the first line of therapy. For cases where medical management is unsuccessful, surgical guidelines and modalities have been developed,^[5]^ including percutaneous trigeminal ganglion radiofrequency thermocoagulation (PRT), gamma knife surgery, and microvascular decompression (MVD). Higher rates of complete pain relief have been reported after PRT, compared to those reported after glycerol rhizolysis or stereotactic radio surgery.^[6,7]^ Moreover, PRT is safe for use in elderly patients and in patients with high surgical risks, and can be repeated if required^[8]^. Because failure and side effects are possible in any kind of surgical modality,^[8,9]^ improvement of medical management is necessary. Moreover, the use psychotropic drugs in patients with TN have not received much attention.

Depression and anxiety are the most notable psychiatric comorbidities associated with TN. Macianskyte et al^[10]^ reported a higher level of anxiety and depression (96.67%) in patients with TN than in patients with atypical facial pain; however, only 26.7% patients with TN used psychotropic drugs. Further, Tolle et al^[11]^ reported concomitant use of medication for anxiety and/or depression in only 33% of patients with TN, and less than 50% patients believed their prescription medications were effective. The treatment of psychiatric comorbidity or emotional distress could be closely related to pain relief. It is important to determine whether pain causes depression or whether depression causes pain in patients with TN. Understanding psychiatric disorders that are associated with TN is necessary and can improve prevention and treatment. However, few studies have exclusively evaluated anxiety and depression disorders in patients with TN.

The primary purpose of this study was to investigate the prevalence of anxiety and depression in patients with TN. We also aimed to identify predictive factors for depression and anxiety, and observe the effect of percutaneous trigeminal ganglion radiofrequency thermocoagulation procedures (PRT) on depression and anxiety in patients with TN.

## 2 Materials and methods

### 2.1 Patients

Patients who were consecutively admitted to the Department of Pain Management, Xuanwu Hospital of Capital Medical University between October 2014 and October 2015 with a diagnosis of classic TN were recruited for this study. Diagnosis of classic trigeminal neuralgia was based on the ICHD-II criteria published by the International Headache Society in 2004 (Headache Classification Subcommittee of the International Headache Society 2004). Symptom duration of ≥3 months and up to at least the week prior to the survey was required. This study was approved by the local Ethics Committee. All patients agreed to participate in the study and provided written informed consent.

Patients with a history of serious or unstable psychological conditions before the first attack of TN, severe motor deficit, difficulty completing the questionnaire, or other sources of pain (lower back pain, fibromyalgia, etc.) were excluded. All patients underwent percutaneous trigeminal ganglion radiofrequency (PRT) operation under intravenous anesthesia as described in a previous publication.^[12]^ After the procedure, intracranial hypotension (low pressure) headache were prevented by putting patients on strict bed rest for 2 days. If their condition remained normal, they were discharged on the third day post-procedure.

### 2.2 Survey questionnaires

The severity of pain was evaluated using a visual analogue scale (VAS) score (from 0, indicating no pain to 10, indicating the worst pain). A 10-cm horizontal line was anchored by 2 descriptors of pain at each end (left end scored 0, i.e. no pain; right end scored 10, i.e. the worst pain imaginable). The patients were asked to mark the point on the line that represented the severity of the pain that they suffered most recently. The VAS score was determined by measuring in millimeters from the left end of the line to the point that the patient marked.

Depression was assessed by the Chinese version of the Beck Depression Inventory-II (BDI), which has good internal consistency and test-retest reliability across various populations, and has been used in China for many years.^[13]^ It contains 21 multiple-choice self-report items to measure depressive symptoms in the past 2 weeks. Each item is scored between 0 and 3, yielding a total BDI score ranging from 0 to 63. Higher total scores indicate more severe depression symptoms. Global BDI scores ranging from 0 to 13 represent “minimal depression,” total scores from 14 to 19 indicate “mild depression,” total scores from 20 to 28 indicate “moderate depression,” and total scores from 28 to 63 indicate “severe depression.” A global BDI score ≥14 is considered positive.

Anxiety was assessed by the Chinese version of the Beck Anxiety Inventory (BAI),^[14]^ which is a 21-item self-report questionnaire, used to measure the severity of anxiety symptoms. Each item is rated on a 4-point Likert-type scale with 0 being “not at all” and 3 being “severely, I could barely stand it.” Total scores range between 0 and 63, with higher scores representing higher severity of anxiety. The BAI is a strong tool for measuring cognitive and somatic aspects of self-reported anxiety symptomatology in both clinical and nonclinical populations. In this study, the Chinese version of the BAI, which has similar psychometric properties, was used to assess the severity of anxiety that TN patients experienced. A BAI score ≥45 is considered positive.

VAS, BDI, and BAI scores were collected at the time of admission and on the day of discharge.

### 2.3 Data collection

All survey questionnaires were completed by an independent interviewer at admission and on the day of discharge. Each item of the questionnaires was explained to the patients before they answered, to ensure accuracy. Demographic and psychosocial backgrounds (gender, age, marital status, education, comorbidities, TN duration and progression, medication and doses, occupation, etc.) were also recorded.

### 2.4 Data analysis

Chi square tests were used to compare BDI and BAI scores according to gender, age, education, marriage status, income, career, comorbidities, pain medicine, affected divisions, and VAS. Student *t*-tests were used to compare the BDI scores and BAI scores before and after radiofrequency treatment. Multiple linear regression analyses were used to predict scores on the BDI and BAI. The criterion for statistical significance was set at *P *<* *0.05. All statistical analyses were completed with the Statistical Package for the Social Sciences, 18.0.0 (SPSS, IBM).

## 3 Results

A total of 167 patients diagnosed with classic TN aged 17 to 91 years were included in this study. The clinical and demographic characteristics of the patients are presented in Table 1.

**Table 1:**
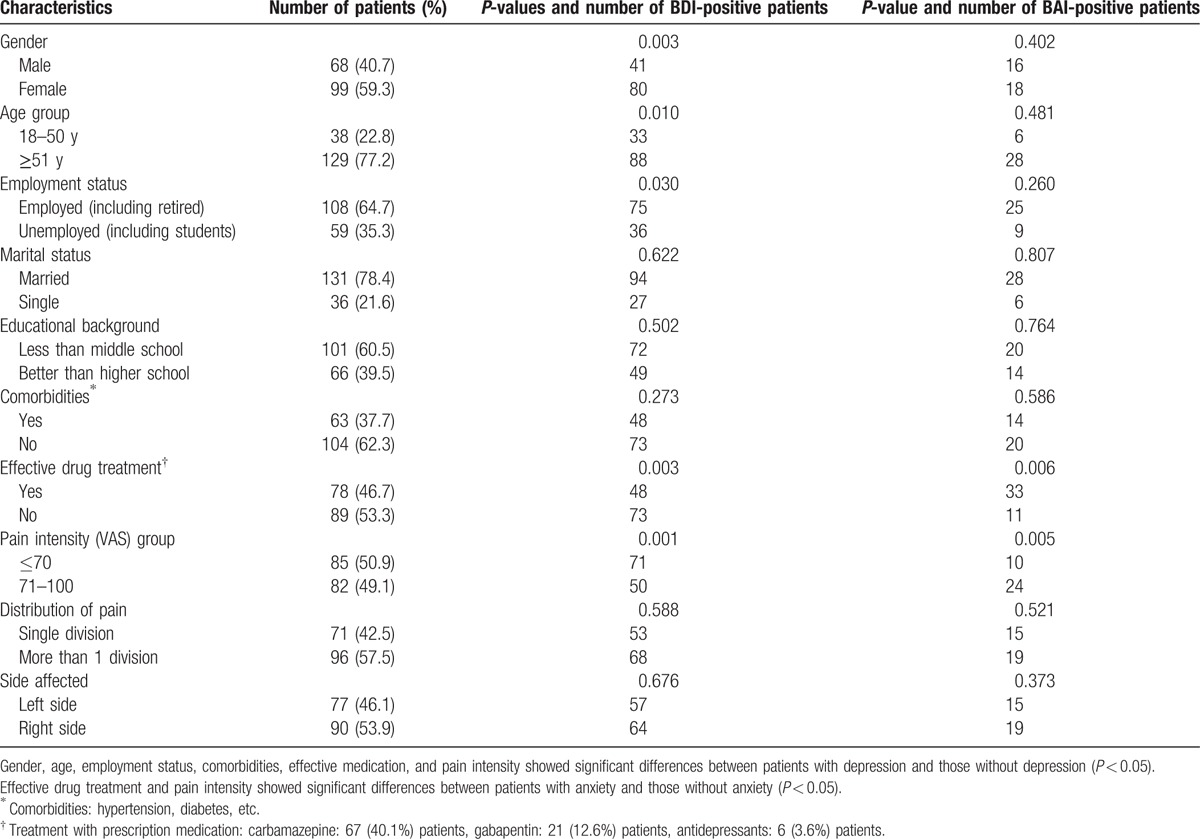
Patient characteristics (N = 167).

### 3.1 Prevalence of anxiety and depression disorders in patients with TN preradiofrequency procedure

121 (72.5%) patients had mild or moderate depression (BD I ≥ 14); 34 patients (20.4%) suffered severe anxiety (BAI ≥ 45); There were significant differences in gender, age, employment status, comorbidities, effective medicine treatment, and pain intensity between patients with depression and those without depression (*P* < 0.05). However, only effective medicine treatment and pain intensity showed significant differences between patients with anxiety and those without anxiety (*P <* 0.05).

### 3.2 Logistic regression analysis of risk factors for anxiety and depression disorders in patients with TN pre-radiofrequency procedure

Results of logistic regression analysis of predictors of depression and anxiety in patients with TN are shown in Table 2. Female sex, age >50 years, ineffective treatment, and pain intensity (VAS ≥7) were significant risk factors for depression. Female patients scored higher on the BDI scales than patients with any other predictor variable; women showed 5.441 times (OR = 5.441) higher prevalence of depression than men did. Patients with high pain intensity (VAS ≥ 7) had 4.052 times (OR = 4.052) higher incidence of depression than did those with VAS<7. Patients older than 50 years had 0.418 times higher prevalence of depression than did patients younger than 50 years, and patients with ineffective treatment showed 1.088 times higher prevalence of depression than did those with effective treatment (*P* < 0.05). There was no significant positive correlation between these risk factors and anxiety (BAI scores; *P* ≥ 0.05).

**Table 2:**
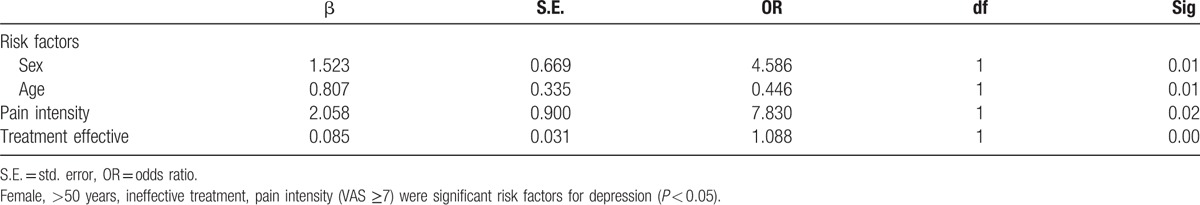
Logistic analysis main factors for depression of patients with trigeminal neuralgia.

### 3.3 Anxiety and depression disorders in patients with TN who achieved pain relief post-radiofrequency procedure

After trigeminal neuralgia radiofrequency, immediate complete pain relief at the time of hospital discharge was reported in 166 (99.4%) patients, in whom VAS scores reduced to ≤3. One patient experienced recalcitrant symptoms, and the VAS score remained 8. The mean BDI and BAI scores of all the patients who experienced pain relief decreased dramatically (Table 3, *P* < 0.05). Thus, pain intensity played an important role in depression and anxiety. On the day of discharge, patients that experienced pain relief had BDI scores ≥14 and BAI scores ≥45. In the patient with recalcitrant pain, the BDI score increased from 3 to 14, but the BAI score decreased from 54 to 35, which indicated that failed operation and pain persistence influenced depression.

**Table 3:**
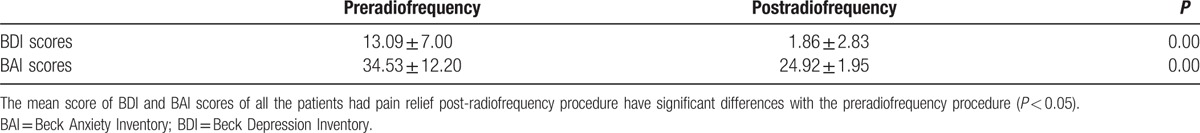
Comparison BDI scores and BAI scores between preradiofrequency and postradiofrequency procedure in patients with trigeminal neuralgia.

## 4 Discussion

The present study elucidated the prevalence of depression and anxiety disorders in 167 patients with intractable TN, who required surgical intervention by PRT. It is the known work to evaluate risk factors for depression and anxiety in patients with TN, and the results may suggest potential strategies for the management of TN with psychosocial intervention. Moreover, we evaluated the effect of successful surgical intervention that alleviated pain on depression and anxiety disorders in patients with TN.

Previous studies have shown that patients with chronic pain are at a higher risk of developing anxiety and depression than is the general population. The incidence of depression in patients with chronic pain is higher (30% to 54%) than that in the general population (2.2%).^[15,16]^ Our results showed a significantly higher prevalence of depression (72.5%) and anxiety (20.4%) in patients with intractable TN. Only 1 previous study has evaluated the prevalence of depression in 30 patients with TN; the incidence of depression reported was 76.7%.^[10]^ Our results supported those from this study, indicating that the prevalence of depression and anxiety in TN is higher with chronic pain. Psychosocial intervention is therefore necessary for patients with TN.

Our patient group was significantly diverse; we could therefore determine predictive factors for depression and anxiety in patients with TN, such as gender, age, employment status, comorbidities, effective medicine treatment, and pain intensity for depression occurrence, and effective medicine treatment and pain intensity for anxiety occurrence. There have been several studies to examine the risk factors for depression and anxiety in patients with pancreatic cancer,^[17]^ breast cancer,^[18]^ coronary heart disease,^[19]^ and rheumatoid arthritis.^[20]^ To our knowledge, this is the first study to determine the predictive factors for depression and anxiety in TN. The results of logistic regression analysis for predictors of depression and anxiety in patients with TN suggested that patients who were female, older than 50 years, or severe pain (VAS > 7) were more prone to depression and anxiety. Surgical failure could also increase BDI score, indicating that failure of pain relief treatment is a predictive factor for depression, as reported by Okamura.^[18]^ Some psychosocial intervention is therefore required for preventing depression and anxiety in patients who only require conservative treatment. Individualized treatment could improve patient outcomes for TN.

Previous studies have shown a positive correlation between the level of depression and pain intensity in patients with chronic pain. Depression and anxiety in such patients may be linked to pain intensity.^[21]^ Thus, severe chronic pain is a likely a risk factor for the development of depression and anxiety. We found that patients with high pain intensity (VAS > 7) had 4.052 times (OR = 4.052) higher incidence of depression than did patients with VAS < 7. Chronic pain is therefore a major predictive factor for depression development. Pain intensity differs from other predictive factors in that it can be alleviated immediately by PRT. We used PRT technology to treat many patients with intractable TN.^[8,9,12]^ Effective PRT surgical could alleviate severe pain and thereby attenuate depression and anxiety induced by TN. Further, we compared pre-operation and post-operation BDI and BAI scores. BDI and BAI scores (and therefore depression and anxiety) decreased dramatically after pain relief, and only 1 patient suffered from pain persistence. Thus, pain intensity is a major predictive factor for depression and anxiety, and the development of depression and anxiety disorders often follow severe pain in TN patients.

The main limitation of this study was that the results were obtained from a group of intractable patients with TN who had to undergo PRT because medication treatments were not efficacious or had intolerable adverse effects. We therefore could not evaluate the combined effects of anticonvulsants and antidepressants. Further, these patients did not represent all patients with TN accurately. In future studies, we will endeavor to recruit a study cohort that is more representative of general patients with TN.

## 5 Conclusion

Given the high incidence of depression and anxiety, psychosocial intervention for patients who are female, older than 50 years, suffer from treatment failure, or experience severe pain (VAS > 7) is necessary, based on the predictive risk factors identified in our study. Depressive and anxiety disorders are accompanied by severe pain, and alleviation of pain by PRT could ameliorate depressive and anxiety disorders.

## References

[1] Dworkin RH Gitlin MJ Clinical aspects of depression in chronic pain patients. Clin J Pain 1991;7:79–94. 1809423 10.1097/00002508-199106000-00004

[2] Bair MJ Robinson RL Katon W Depression and pain comorbidity: a literature review. Arch Intern Med 2003;163:2433–45. 14609780 10.1001/archinte.163.20.2433

[3] Castro M Kraychete D Daltro C Comorbid anxiety and depression disorders in patients with chronic pain. Arq Neuropsiquiatr 2009;67:982–5. 20069205 10.1590/s0004-282x2009000600004

[4] Kayhan F Albayrak GI Kayhan A Mood and anxiety disorders in patients with chronic low back and neck pain caused by disc herniation. Int J Psychiatry Clin Pract 2016;20:19–23. 26524007 10.3109/13651501.2015.1100314

[5] Cruccu G Aziz TZ Garcia-Larrea L EFNS guidelines on neurostimulation therapy for neuropathic pain. Eur J Neurol 2007;14:952–70. 17718686 10.1111/j.1468-1331.2007.01916.x

[6] Lopez BC Hamlyn PJ Zakrzewska JM Systematic review of ablative neurosurgical techniques for the treatment of trigeminal neuralgia. Neurosurgery 2004;54:973–82. 982–983. 15046666 10.1227/01.neu.0000114867.98896.f0

[7] Taha JM Tew JJ Comparison of surgical treatments for trigeminal neuralgia: reevaluation of radiofrequency rhizotomy. Neurosurgery 1996;38:865–71. 8727810 10.1097/00006123-199605000-00001

[8] Tang YZ Jin D Li XY Repeated CT-guided percutaneous radiofrequency thermocoagulation for recurrent trigeminal neuralgia. Eur Neurol 2014;72:54–9. 24853911 10.1159/000357868

[9] Lai GH Tang YZ Wang XP CT-guided percutaneous radiofrequency thermocoagulation for recurrent trigeminal neuralgia after microvascular decompression: a cohort study. Medicine (Baltimore) 2015;94:e1176. 26266350 10.1097/MD.0000000000001176 PMC4616680

[10] Macianskyte D Januzis G Kubilius R Associations between chronic pain and depressive symptoms in patients with trigeminal neuralgia. Medicina (Kaunas) 2011;47:386–92. 22112988

[11] Tolle T Dukes E Sadosky A Patient burden of trigeminal neuralgia: results from a cross-sectional survey of health state impairment and treatment patterns in six European countries. Pain Pract 2006;6:153–60. 17147591 10.1111/j.1533-2500.2006.00079.x

[12] Tang YZ Wu BS Yang LQ The long-term effective rate of different branches of idiopathic trigeminal neuralgia after single radiofrequency thermocoagulation: a cohort study. Medicine (Baltimore) 2015;94:e1994. 26559288 10.1097/MD.0000000000001994 PMC4912282

[13] Wu PC Huang TW Gender-related invariance of the Beck Depression Inventory II for Taiwanese adolescent samples. Assessment 2014;21:218–26. 22517921 10.1177/1073191112441243

[14] Chen HM Wang HH Chiu MH Effectiveness of a releasing exercise program on anxiety and self-efficacy among nurses. West J Nurs Res 2016;38:169–82. 25326004 10.1177/0193945914555405

[15] Dworkin RH Hetzel RD Banks SM Toward a model of the pathogenesis of chronic pain. Semin Clin Neuropsychiatry 1999;4:176–85. 10498785 10.153/SCNP00400176

[16] Fekadu A Alem A Medhin G Utility of the concept of minor depressive disorder: evidence from a large rural community sample in a developing country setting. J Affect Disord 2007;104:111–8. 17448542 10.1016/j.jad.2007.03.008

[17] Akizuki N Shimizu K Asai M Prevalence and predictive factors of depression and anxiety in patients with pancreatic cancer: a longitudinal study. Jpn J Clin Oncol 2016;46:71–7. 26590013 10.1093/jjco/hyv169

[18] Okamura H Watanabe T Narabayashi M Psychological distress following first recurrence of disease in patients with breast cancer: prevalence and risk factors. Breast Cancer Res Treat 2000;61:131–7. 10942098 10.1023/a:1006491417791

[19] Tully PJ Cosh SM Baumeister H The anxious heart in whose mind? A systematic review and meta-regression of factors associated with anxiety disorder diagnosis, treatment and morbidity risk in coronary heart disease. J Psychosom Res 2014;77:439–48. 25455809 10.1016/j.jpsychores.2014.10.001

[20] Ryan S McGuire B Psychological predictors of pain severity, pain interference, depression, and anxiety in rheumatoid arthritis patients with chronic pain. Br J Health Psychol 2016;21:336–50. 26525312 10.1111/bjhp.12171

[21] Altindag O Gur A Altindag A The relationship between clinical parameters and depression level in patients with myofascial pain syndrome. Pain Med 2008;9:161–5. 18298698 10.1111/j.1526-4637.2007.00342.x

